# Tafamidis reduces disease progression in patients with transthyretin familial amyloid polyneuropathy: supportive post-hoc analyses of a pivotal trial

**DOI:** 10.1186/1750-1172-10-S1-P11

**Published:** 2015-11-02

**Authors:** Denis Keohane, Jeffrey Schwartz, Balarama Gundapaneni, Michelle Stewart, Leslie Amass

**Affiliations:** 1 Pfizer, Global Innovative Products, NY 10017, New York, USA; 2 Pfizer, Global Innovative Products-Statistics, CT 06340, Groton, USA; 3 inVentiv Health, Statistician, MA 01803, Burlington, USA; 4 Pfizer, Specialty Care Medicines Development Group, CT 06340, Groton, USA; 5 Pfizer, Global Innovative Pharma Business, PA 19426, Collegeville, USA

## Background

Safety and efficacy of once-daily 20 mg tafamidis, a transthyretin (TTR) stabilizer, was evaluated in an 18-month, multicentre, randomized, double-blind, placebo-controlled study in 128 patients with early symptomatic V30M TTR familial amyloid polyneuropathy (TTR-FAP). In the intent-to-treat population, a responder analysis for Neuropathy Impairment Score-Lower Limb (NIS-LL) (co-primary with Norfolk Quality of Life-Diabetic Neuropathy) favoured tafamidis (P=0.07). A pre-specified, key secondary analysis of change from baseline to Month 18 in NIS-LL continuous scores was significant (P=0.04). Placebo-corrected point estimates for 5 pre-specified and validated measures of disease progression also favoured tafamidis and were directionally consistent. Additional post-hoc analyses supporting tafamidis for delaying progression of TTR-FAP are reported here.

## Methods

Change from baseline in NIS-LL over time was analysed with the addition of the baseline values as a covariate in a repeated measures model. A sensitivity multiple imputation analysis with imputed values based on assigned treatment group was also performed. Additionally, change in NIS-LL+ summated 7 (neurophysiological function composite endpoint) over time was assessed.

## Results

When adjusted for baseline NIS-LL disease severity, statistical significance in change from baseline to Month 18 NIS-LL was retained. The magnitude of separation between placebo and tafamidis was consistent across the full range of baseline values (least squares mean difference = 2.7 points; 95% confidence interval [CI]: 0.1, 5.2; P<0.05). Treatment effect estimates from the multiple imputation analysis, although reduced, were similar to those from the analysis of change from baseline to Month 18, and remained significant. With each batch run representing the results combined from 1000 multiply imputed data sets, the difference in NIS-LL change from baseline to Month 18 for tafamidis versus placebo was -2.787 (standard error [SE]: 1.345; 95% CI: -5.423, -0.151; P=0.04) for Batch Run 1; for Batch Run 2, the difference was -2.815 (SE: 1.351; 95% CI: -5.464, -0.166; P=0.04); and for Batch Run 3, the difference was -2.798 (SE: 1.347; 95% CI: -5.438, -0.157; P=0.04). For NIS-LL+ summated 7, at Month 6, the mean change (standard deviation) was 1.8 (4.82) for the tafamidis group and 4.0 (7.12) for the placebo group (P=0.053); while significant differences between treatment groups were observed at Months 12 (1.6 [5.27] vs 7.8 [8.87], P=0.00009) and 18 (3.4 [5.95] vs 8.8 [10.44], P=0.0043).

## Conclusions

The beneficial effects of tafamidis in delaying neurological impairment in TTR-FAP are further supported by these post-hoc analyses.

## Note

Note: First presented at the 1st Congress of the European Academy of Neurology, Berlin, Germany, June 2015

